# "Original Antigenic Sin" in SARS-CoV-2 Vaccination Followed by Infection

**DOI:** 10.7759/cureus.32548

**Published:** 2022-12-15

**Authors:** Yandy M Castillo-Aleman, Carlos A Villegas-Valverde, Yendry Ventura-Carmenate, Gisela M Suarez-Formigo, Antonio A Bencomo-Hernandez

**Affiliations:** 1 Department of Immunology, Abu Dhabi Stem Cells Center, Abu Dhabi, ARE; 2 Laboratory of Immunology, Abu Dhabi Stem Cells Center, Abu Dhabi, ARE

**Keywords:** vaccine, sars-cov-2, covid-19, antigenic variation, adverse events

## Abstract

Although the *"original antigenic sin"* (OAS) effects have been predicted against new variants of severe acute respiratory syndrome coronavirus 2 (SARS-CoV-2), only a few pieces of evidence are available regarding its impact on the safety and effectiveness of coronavirus disease 2019 (COVID-19) vaccines. This article aims to provide an immunological explanation for the delayed side effects of a SARS-CoV-2 vaccine during an episode of natural infection. We reported a case of a 39-year-old male healthcare worker who complained about pruritus and discomfort around the injection site of an inactivated SARS-CoV-2 vaccine administrated 18, 17, and 13 months earlier. Those symptoms resembled the side effects previously experienced with one of the booster doses, and a sole erythematous papule was also documented. The patient was diagnosed with COVID-19 one or two days after noticing these local signs and symptoms, and high serum titers of immunoglobulin M (IgM) and immunoglobulin E (IgE) were found five weeks after the onset, along with SARS-CoV-2-specific immunoglobulin G (IgG) antibodies. Therefore, the OAS might be a plausible phenomenon to consider in individuals immunized with inactivated vaccines and exposed secondarily to a wild virus with antigenic variations.

## Introduction

Severe acute respiratory syndrome coronavirus 2 (SARS-CoV-2) infection emerged in the current coronavirus disease 2019 (COVID-19) pandemic, which has triggered a notable development of SARS-CoV-2 vaccines. Consequently, there have been considerable achievements in understanding the humoral and cellular immune responses following vaccination and natural infection. As most vaccines use a two-dose prime-boost approach to generate an immune response against SARS-CoV-2 antigens, individuals with a previous infection develop higher antibody titers than uninfected ones [1].

However, the *"original antigenic sin"* (OAS) effect at the immune system’s second exposure to a similar pathogen to which it was previously exposed, generating cross-reactive antibodies already present against the initial antigens, has been predicted against new variants of SARS-CoV-2 [2]. Nevertheless, prior viral exposure (SARS-CoV-2 vaccine-induced or wild/natural infection) impacts subsequent viral exposures (via vaccination or natural infection) positively or negatively [3].

Since massive vaccination has been implemented, reports of adverse events following approved vaccines have increased worldwide. The most frequent cutaneous effects reported are localized at the injection site [4], with a particular interest in the “COVID arm” phenomenon, a harmless delayed hypersensitivity reaction mostly related to mRNA vaccines [5] while those events classified as delayed local reactions typically start eight days or more after vaccination [4].

We report significantly delayed side effects of an inactivated SARS-CoV-2 vaccine during an episode of natural infection in a case where the OAS is a plausible phenomenon. This paper aims to provide an immunology-based explanation of the clinical events reported and raises the emerging need to evaluate further the long-term safety and effectiveness profiles of current COVID-19 vaccines that received an emergency use or conditional marketing authorization.

## Case presentation

A 39-year-old male healthcare worker diagnosed with mild asthma received three doses of an inactivated SARS-CoV-2 vaccine (BBIBP-CorV; Sinopharm, China) at 18, 17, and 13 months preceding his COVID-19 infection. Each vaccine was given intramuscularly (0.5 mL) in the right deltoid area; the second and third doses were associated with transient itchiness, redness (third dose only), and discomfort around the injection site with spontaneous resolution within 24-48 hours. His vaccination record showed the hepatitis B vaccine four years before, and no other vaccines followed SARS-CoV-2 immunization.

**Figure 1:**
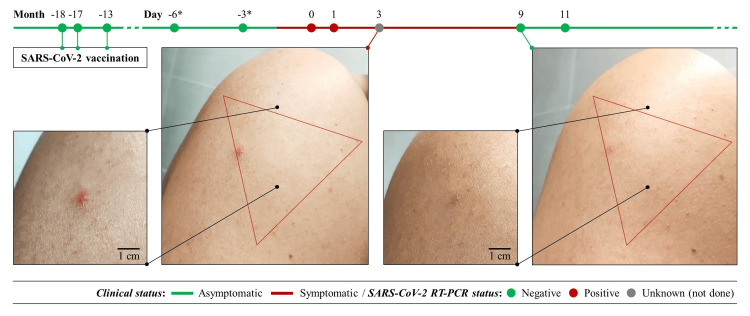
Erythematous papule at the injection site Day 0 was considered the first day of a SARS-CoV-2-positive RT-PCR nasopharyngeal swab; the triangle represents the recommended injection site in the deltoid muscle RT-PCR: Real-Time Polymerase Chain Reaction (*) Testing due to close and continuous contact with a confirmed COVID-19 patient on days -10 and -9

After eight or nine days of being in close and continuous contact with a confirmed COVID-19 case, the patient started with pruritus and discomfort around the vaccine administration site, resembling the side effects experienced previously. The difference in this episode consists of the appearance of one sole erythematous papule, firm, slightly indurated, around 0.5-0.6 cm in diameter in the SARS-CoV-2 vaccine injection area, as shown in Figure 1 (grade 1 vaccination complication, Common Terminology Criteria for Adverse Events - CTCAE v.5.0). Intermittent headache, body pain, and mild asthma attacks were associated, and he was diagnosed with COVID-19 after one or two days of onset of the cutaneous features.

Perilesional tenderness and pruritus lasted until Days 2-3 without warmth or lymphadenopathy, and the cutaneous events were solved spontaneously within one week, without complications. The complete blood counts before and after the COVID-19 infection were unremarkable. However, high serum titers of IgM (361.2 mg/dL, reference range 40 - 230 mg/dL) and IgE (344.9 ng/mL, reference value <240 ng/mL) were found five weeks after the onset of COVID-19 symptoms. At that time, anti-SARS-CoV-2 total antibodies were also reactive (153, positive >1.1 index), along with SARS-CoV-2-specific IgG antibodies (>200 BAU/mL, positive >17.8 BAU/mL).

## Discussion

It is known that the OAS phenomenon occurs in the second exposure to a similar pathogen (SARS-CoV-2 wild-type virus) to which it has previously been exposed (inactivated SARS-CoV-2 vaccine). Herein, overproduction of memory B cells could affect the naïve B cells activation capable of generating efficient antibodies, triggering immune evasion of the novel variants [2] that emerged after one year of the COVID-19 pandemic.

As reported by Kostoff et al., numerous long-term potential issues concerning vaccines have been identified that can explain [3], at least partially, these side effects. The distinct genetic variants (antigenic distance hypothesis) [3] along with the viral exposure and expected decline in titers of neutralizing antibodies [5] have resulted in the reported infection, even though the symptoms were not severe.

Antibodies originating from the inactivated vaccine may have specificity or cross-reactivity with immunodominant antigens circulating (or viremia) preceding virus detection on the nasopharyngeal swab. Irrespective of the viral variant causing the primary response, imprinting SARS-CoV-2 specific antibody reactions lead to a secondary response that favorably boosts against the primary antigen [6]. Moreover, cross-reactivity between seasonal coronaviruses and SARS-CoV-2 has also been documented [7], resulting in OAS effects in patients with pre-existing immunity against related coronaviruses.

Park et al. stated that the OAS might cause additional inflammatory responses in specific circumstances of antigenic variation [8]. After inactivated whole virus vaccines are administered (with limited antigen availability), the immune repertoire freezes, and memory B and CD4 T cell responses are skewed toward previously exposed epitopes [8]. We hypothesized that the winner-take-all dominant epitope effects could have simulated the local adverse events noticed on vaccination one year ago.

Whereas the Th17 role has been linked to an enhanced immune response producing eosinophilic pulmonary immunopathology in COVID-19 [9], the previous history of asthma and the vaccine adjuvant (aluminum-hydroxide) could have diminished these effects by polarizing into Th2 responses [4,9]. Eosinophilia was not verified during the first days, but IgE titers remained elevated five weeks after the onset of the symptoms.

Additionally, at least one vaccine was given borderline to the recommended area in this case, which could have modified the antigenic depot exerted by alum adjuvants in the vaccine-site microenvironment, which in association with the effects described earlier, might have generated tertiary lymphoid structures (TLS). When analyzing the adverse events at the vaccination site, allergic relationship, Arthus phenomenon, and inflammation due to a TLS induction require a differential diagnosis. The first two hypersensitivity reactions commonly occur immediately, being ruled out in our case. Therefore, TLS reacting to successive cognate antigenic challenges in the OAS scenario is a plausible explanation. The stimulus caused by the infection may unleash the production of antibodies against the SARS-CoV-2 vaccine antigens, and they respond in the tertiary lymphoid follicles [10].

Although an increase in the number of reports of skin manifestations has been documented in the context of SARS-CoV-2 infection and, more recently, after vaccination against COVID-19 [4], chance influence cannot be ruled out so we described these effects as adverse events, not reactions. The immunopathogenic mechanisms and the causal association can only be theorized, but the accumulated scientific evidence supports our suspicions. Despite our limitations related to the nature of a single case report and the lack of histology and comprehensive immune profiling, this case report can shed light on future research on vaccination against SARS-CoV-2, natural infection, and its cutaneous manifestations in the OAS context. It might be pertinent as per the mass immunization programs, the incidence of COVID-19, and emerging new variants worldwide.

## Conclusions

The OAS is a plausible phenomenon to consider in individuals immunized with inactivated vaccines and exposed secondarily to a wild virus with antigenic variations. Previous immunization status, vaccine type, site of injection, the timing between exposures, antigenic variations, and individual immune competency, among other factors, shall be considered in understanding the immune response against SARS-CoV-2. Consequently, there is an emerging need to evaluate further the long-term safety and effectiveness of COVID-19 vaccines against new variants and outcomes of natural infections.
